# Electrochemical and Morphological Investigations of Ga Addition to Pt Electrocatalyst Supported on Carbon

**DOI:** 10.1155/2017/8786013

**Published:** 2017-03-29

**Authors:** Giordano T. Paganoto, Deise M. Santos, Tereza C. S. Evangelista, Marco C. C. Guimarães, Maria Tereza W. D. Carneiro, Josimar Ribeiro

**Affiliations:** ^1^Departamento de Química, Centro de Ciências Exatas/UFES, Av. Fernando Ferrari, 514 Goiabeiras, 29075-910 Vitória, ES, Brazil; ^2^Diretoria de Pós-Graduação, Instituto Federal do Espírito Santo (IFES), Av. Rio Branco, 50 Santa Lúcia, 29056-255 Vitória, ES, Brazil; ^3^Departamento de Morfologia, Centro de Ciências da Saúde, UFES, Campus Maruípe, 29049-999 Vitória, ES, Brazil

## Abstract

This paper is consisted in the synthesis of platinum-based electrocatalysts supported on carbon (Vulcan XC-72) and investigation of the addition of gallium in their physicochemical and electrochemical properties toward ethanol oxidation reaction (EOR). PtGa/C electrocatalysts were prepared through thermal decomposition of polymeric precursor method at a temperature of 350°C. Six different compositions were homemade: Pt_50_Ga_50_/C, Pt_60_Ga_40_/C, Pt_70_Ga_30_/C, Pt_80_Ga_20_/C, Pt_90_Ga_10_/C, and Pt_100_/C. These electrocatalysts were electrochemically characterized by cyclic voltammetry (CV), chronoamperometry (CA), chronopotentiometry (CP), and electrochemical impedance spectroscopy (EIS) in the presence and absence of ethanol 1.0 mol L^−1^. Thermogravimetric analysis (TGA), energy dispersive X-ray spectroscopy (EDX), X-ray diffraction (XRD), and transmission electron microscopy (TEM) were also carried out for a physicochemical characterization of those materials. XRD results showed the main peaks of face-centered cubic Pt. The particle sizes obtained from XRD and TEM analysis range from 7.2 nm to 12.9 nm. The CV results indicate behavior typical of Pt-based electrocatalysts in acid medium. The CV, EIS, and CA data reveal that the addition of up to 31% of gallium to the Pt highly improves catalytic activity on EOR response when compared to Pt_100_/C.

## 1. Introduction

A lot of the environmental damage caused by human action is related to the use of fossil fuels combined with inefficient energy converters. In this scenario, due to the growing concerns on climate change issues, the fuel cell technology is a promising energy source for transportation, stationary, and portable applications [1], especially the* proton exchange membrane fuel cell* (PEMFC), due to its low temperature operation, high density power, easy scale-up, and low pollutant emission [2].

Throughout the development of PEMFCs, low molecular weight alcohols, particularly methanol and ethanol, have also been tested as alternative fuel due to their liquid state (at room temperature) that allows easy handling, high mass density energy, and the fact that they can be directly injected into the cell [3]. In Brazil, the well-established bioethanol production industry makes the direct alcohol fuel cells (DAFCs), especially the direct ethanol fuel cells (DEFCs), a very promising power source [4–6]. The complete electrochemical oxidation of ethanol can yield 12e^−^ for energy conversion, according to reaction (1). However, the C-C bound cleavage is very difficult to perform at low temperature, and main reaction products are acetaldehyde and acetic acid in acid medium or acetate in alkali medium [7].

(1) $$\begin{array}{c} {CH}_{3}{CH}_{2}OH+3H_{2}O⟶2{CO}_{2}+12H^{+}+12e^{-} \end{array}$$

The Pt-based electrocatalysts are the best material known so far, able to promote the dissociative adsorption of small organics molecules, such as methanol, ethanol, and glycerol [8]. Nevertheless, the difficulty to break C-C bond provides two-carbon intermediates during the ethanol oxidation reaction (EOR) on Pt-based materials. Furthermore, problems such as anodic poisoning due to a strong adsorption of CO and CH_x_ species and electrocatalyst degradation lead to an efficiency loss of the cell [9, 10]. Thus, new materials, for example, Pt-Sn [11], Pt-Ni [11], Pt-Rh [12], Pt-Ru [13], Pt-Ir [14], and Pt-Co [14] binary alloys and different ternary alloys [11, 15–18], have been studied to enhance CO tolerance and promote CO_2_ formation.

The presence of different metals in Pt structure, even if in low quantity, might supply the active sites of Pt with oxygen in alcohol electrochemical oxidation reactions due to oxygenated species generated, avoiding the poisoning of Pt sites by CO, increasing the electrocatalytic activity [18]; this behavior is reported in Pt-Ru and Pt-Sn electrocatalysts, due to CO adsorption energy decrease and bifunctional effect [19]. Moreover, according to Pearson's acid-base theory [20], Pt is considered a soft acid and the carbonyl group is a soft base, so there is a strong interaction between them. The presence of other metals in oxygenated form could promote less CO adsorption, according to its oxidation state.

Recently, gallium and its alloys have attracted considerable interest as electrode materials because of their low toxicity and melting point (29.8°C) [21, 22]. Metallic gallium can be easily incorporated into microscale channels [23].

Gallium is a metallic element in Group 13 of the periodic table. Solid gallium has an orthorhombic crystal structure and displays a conchoidal fracture similar to glass. Gallium has the oxidation states of +1 and +3 valences. The element rapidly dissolves in either aqua regia or concentrated sodium hydroxide in an aqueous medium. The element is suited to readily form alloys (e.g., eutectic alloys) with most metals in conjunction with being a component in low-melting alloys [24]. It is well known that metallic gallium is in self-passivation by an ultrathin gallium oxide layer upon exposure to air, behavior similar to aluminum [22].

The paper proposed by Hogarth and Ralph [25] reported that the PtGa/C electrocatalyst showed a promotional effect toward methanol oxidation reaction in acid electrolyte, reducing the alcohol oxidation potential when compared Pt, PtOs, PtPd, PtIr, PtW, and PtRh. Kumar et al. [26] recently found that the PtGa/graphene showed better activity for methanol oxidation reaction and oxygen oxidation reaction than a commercial Pt/C catalyst. PtGa alloy decreased the CO electrooxidation potential in 0.2 V versus ERH compared to pure platinum. Despite the indications of good catalytic activity of PtGa in methanol electrooxidation, very scarce information is found for renewable alcohol such as ethanol, and no more references about PtGa/C materials for electrocatalysis were found in the pertinent literature. Thus, this paper reports a systematic physicochemical and electrochemical investigation of binaries PtGa/C electrocatalysts toward ethanol oxidation reaction. The results show that the addition of Ga in the Pt-based electrocatalysts improves EOR when compared to a pure Pt/C electrocatalyst.

## 2. Experimental Section

The first procedure was the acid treatment of carbon Vulcan XC72 (Carbot), consisting of washing 2 g in boiling water at 100°C for 30 minutes and then washing it again with water at room temperature. After this stage, the water was withdrawn and the carbon was placed in a warm H_2_SO_4_ solution (Sigma-Aldrich) 1.0 mol L^−1^ for 30 minutes. After performing the functionalization step (previous step) with sulfuric acid, the carbon was dried at 100°C and annealed at 400°C for 1 hour in air atmosphere. Both procedures were performed in oven QUIMIS Q318M model.

PtGa/C electrocatalysts supported on Vulcan carbon XC-72 were prepared through thermal decomposition of polymeric precursors method [15, 17, 27–29]. This method consists on the synthesis of resins from metal precursors (metal salt) and citric acid (Merck) in a mixture with ethylene glycol (Merck), at a molar ratio of 1 : 4 : 16, respectively. After the dissolution of citric acid in ethylene glycol (at 60–65°C), the metal precursor (H_2_PtCl_6_, Ga(NO_3_)_3_, Sigma-Aldrich) dissolved previously in alcohol (0.05 mol L^−1^) and isopropanol (Merck) for platinum and ethanol (Sigma-Aldrich) for Gallium, was slowly added. After total dissolution of the metal precursor, the temperature of mixture was increased to 85–90°C and kept under stirring for 1-2 h for the esterification step. Finally, the metal concentration for each resin was determined using inductively coupled plasma (ICP-OES model optima 7300 V), which were Pt = 5512 mg L^−1^ and Ga = 7311 mol L^−1^.

PtGa/C electrocatalysts consisting of 40 wt.% metal and 60 wt.% carbon were prepared by mixing the appropriate amounts of each metal resin together. Then, the electrocatalysts were dispersed in 1 mL ethanol (Sigma-Aldrich) for 20 minutes, in ultrasonic bath (Thornton T14 model). The solvent was evaporated in an oven at 80°C and then annealed at 350°C for 3 hours in oven (Model QUIMIS Q318M model). Six PtGa/C electrocatalysts were prepared, with the following nominal compositions: Pt_50_Ga_50_/C, Pt_60_Ga_40_/C, Pt_70_Ga_30_/C, Pt_80_Ga_20_/C, Pt_90_Ga_10_/C, and Pt_100_/C.

The physicochemical characterization of the material was carried out by transmission electronic microscopy (TEM) on a JEOL/JEM-1400 microscope operating at 200 kV. Scanning electronic microscopy coupled with energy dispersive X-ray spectroscopy (SEM-EDX) was performed on a Carl Zeiss microscope EVO10 model. Thermogravimetric analysis (TGA) using a TA instrument SDT Q600 model V20.9 build 2.0, in dry air, at a heating rate of 5°C/minute from 25 to 550°C was accomplished for the resins and carbon. Two isotherms at 70 and 350°C were applied for the precursor catalyst solution, for 10 minutes and 180 minutes, respectively. For the X-ray diffraction (XRD) analysis was performed on a Bruker D8 advance using CuK*α* radiation (*λ* = 1.5406 Angstrom). The following parameters were kept constant throughout the tests: 2*θ* = 10° to 90°, step = 0.01°. Average crystallite size, *d*, was estimated by means of the Scherrer equation [30], where *λ* is the radiation wavelength, *B* is the reflection width at half-maximum intensity (FWHM), and*θ* is the angle at the maximum intensity:

(2) $$\begin{array}{c} d=\frac{0.9\times \lambda }{B\times \cos\left(\theta \right)}. \end{array}$$

### 2.1. Electrochemical Investigations

All the solutions employed in this paper were prepared with 18 MΩ cm water produced and purified in a Reverse Osmosis System (Puritech model RO 300). Electrochemical studies were conducted by using 0.5 mol L^−1^ H_2_SO_4_ (Sigma-Aldrich) as supporting electrolyte. The electrochemical experiments were carried out in a one-compartment cell with a main body of 50 cm^3^. The working electrode was prepared with an ink consisting of 5 mg of the catalyst, 25 *μ*L Nafion® (Sigma-Aldrich), and 475 *μ*L ethanol. The solution was then placed in an ultrasonic bath for 20 minutes, followed by deposition of 100 *μ*L from a micropipette onto a graphite carbon substrate with 0.16 cm^2^ of geometric area freshly polished with alumina (0.3 *µ*m) and dried in an oven at 60°C for 20 minutes. The graphite carbon electrode was used as a counterelectrode and a Reversible Hydrogen Electrode (RHE) was used as a reference electrode, which was positioned close to the working electrode. The electrochemical activity of the electrocatalysts was assessed by cyclic voltammetry (CV) in acid medium, from 0.05 to 1.2 V versus RHE, at scan rate of 50 mV s^−1^, in the presence and absence of ethanol (Sigma-Aldrich) 1.0 mol L^−1^. Freshly prepared working electrodes were submitted to 50 cycles of CV to promote a deep activation of the electrocatalysts; this procedure furnished a steady-state condition for voltammetric curves.

The chronoamperometry (CA), in the presence of ethanol (Sigma-Aldrich) 1.0 mol L^−1^, was measured at 400 mV versus RHE for 2 hours. Current densities of 3.0 mA cm^−2^ were applied for 15 hours at chronopotentiometry experimental (CP). The electrochemical impedance spectroscopy (EIS) was carried out in ethanol 1.0 mol L^−1^ at 0.4 V on a frequency range of 100 kHz to 5 mHz in the single sine mode with an amplitude of 5 mV p/p. The electrochemical experiments were performed using an AUTOLAB potentiostat/galvanostat 302N model.

## 3. Results and Discussion

### 3.1. Physicochemical Characterizations

**Figure 1:**
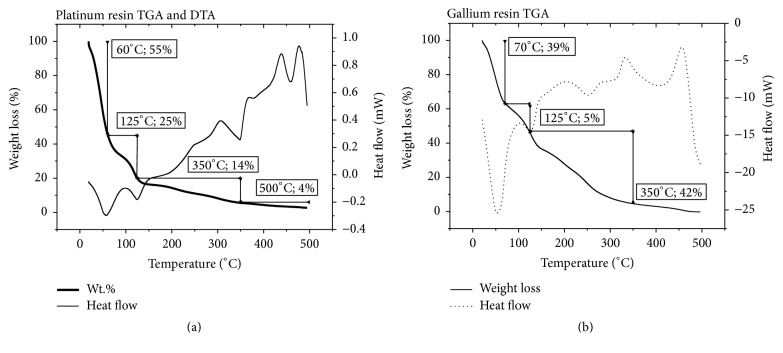
TGA curves obtained for (a) gallium and (b) platinum resins at heating rate of 5°C min^−1^ from room temperature to 500°C.

Thermogravimetric analysis of the resin helped in monitoring the setting of a temperature range for the catalyst calcination (between 350 and 550°C), in which large variation in the mass of resin is not observed and all organic compounds had been decomposed. The representative TGA and DTA curves obtained for platinum and gallium resins are presented in Figures 1(a) and 1(b), respectively. With this technique, one can follow the weight loss and temperature difference of the resin as a function of the temperature. The results show that there is huge weight reduction at ca. 70°C, with total weight loss of 60% for platinum and 39% for gallium resin, associated mainly with the evaporation of the solvent, isopropanol, and ethylene glycol. Moreover, there are two other weight loss processes: one set at ca. 125°C and the second located at ca. 245°C, which continues until ca. 350°C. These processes are associated with the thermal decomposition of the polymeric resin. Thereafter, the weight remains practically constant that suggests that all the organic compounds have been removed and the residual material has a stable and fixed composition.

**Figure 2:**
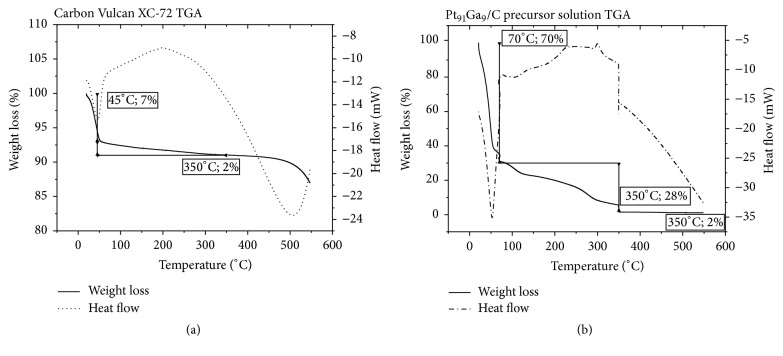
TGA and DTA curves obtained for (a) carbon Vulcan XC-72 and for (b) precursors solution of Pt_91_Ga_9_/C electrocatalyst with isotherm at 70°C and 350°C, for 10 min and 180 min, respectively, both at heating rate of 5°C min^−1^ from room temperature to 550°C.

The representative TGA and DTA curves obtained for carbon Vulcan XC-72 after acid treatment are presented in Figure 2(a). The carbon Vulcan XC-72 TGA curve displays insignificant weight loss during the heating process until 350°C, with the 7% weight loss until 45°C possibly being related to humidity loss in an endothermic process and the 2% weight loss from 45 to 350°C, in an exothermic process, possibly attaching to combustion of the carbon itself or the residual sulfur composts of the acid treatment. Moreover, to ensure the electrocatalyst composition, a TGA analysis of precursor solution of the Pt_91_Ga_9_/C electrocatalyst was performed as described in Experimental Section and its results are shown in Figure 2(b). As observed for the TGA of the resins, the precursor solution of the catalyst Pt_91_Ga_9_/C electrocatalyst also showed an endothermic process below 70°C, indicating evaporation of the solvents, ethanol and isopropanol, representing a loss of 70% weight. The same weight loss behavior (59.91%) was observed by [27] in a TGA analysis of a precursor solution of a quaternary Pt_60_Sn_10_Ni_10_Ir_20_ electrocatalyst supported on Vulcan XC-72 carbon conducted under similar conditions. After the isotherm at 70°C it is possible to observe a continuous weight loss (29%) which refers to the combustion of organic compounds that compose the resin as seen in the TGA of the resins. As expected, after the isotherm being at 350°C for three hours, there was no weight loss or variation of heat flow indicating that the electrocatalyst has a fixed and stable composition after calcination, as observed before.

Nominal and experimental compositions of all electrocatalysts are very close to each other according to energy dispersive X-ray spectroscopy (EDX) in Table 1, the exception being Pt_60_Ga_40_/C electrocatalyst, that showed distinct nominal composition from experimental Pt_69_Ga_31_/C. It is observed that all electrocatalysts have a significant amount of oxygen (from 3.4 to 10.3%), which can be related to the formation of metal oxides (PtO_x_ and/or Ga_2_O_3_). The oxygenated groups resulted from the combustion of the precursor resins in air atmosphere.

**Figure 3:**
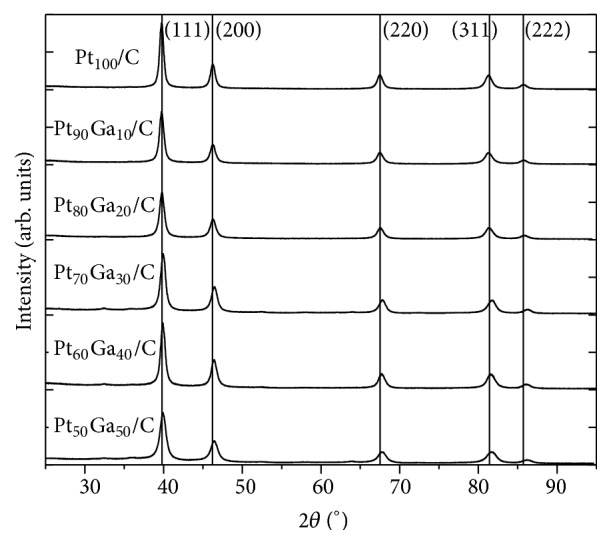
XRD pattern of Pt-based electrocatalysts prepared through thermal decomposition of polymeric precursors method obtained with CuK*α* radiation (*λ* = 1.5406 Å) and step rate = 0.01° min^−1^.

Figure 3 shows the X-ray diffraction pattern of the PtGa/C electrocatalysts, in which vertical lines mark the diffraction peaks from the standard (PDF-01-087-0646) [31]. The electrocatalysts exhibit peaks at diffraction angles on values 2*θ* = 39.8°; 46.2°; 67.5°; 81.4°; and 85.7° concerning the respective diffraction planes (111), (200), (220), (311), and (222) of platinum with face-centered cubic structure (FCC). Despite having a significant amount of oxygen in the composition of the electrocatalysts, crystalline formation of the platinum or gallium oxides was not identified; however this hypothesis cannot be dismissed because the oxides might be present in amorphous form, since the gallium oxide has well defined structure upon heating above 500°C [32] and platinum oxides are well formed at 900°C [33].

The lattice parameters were obtained using Bragg's law and the average crystallite sizes for different crystallographic planes were estimated through Scherrer equation [30]. The results are shown in Table 2.

It is observed that all electrocatalysts have smaller lattice parameters than the platinum catalyst (*a* = 3.922 Å), indicating contraction of the crystal lattice, probably due to the incorporation of gallium in the structure of platinum, since the lattice parameter decreases from 3.922 to 3.906 Å as the experimental amount of gallium increases up to 31%. The addition of metal with atomic radius smaller than that of platinum to its cubic structure, as Ni, Ru, Ir, or Rh, with atomic radius of 124, 134, 135, and 134.5 pm, respectively, promotes a decrease in the lattice parameters indicating a contraction of the crystal lattice of platinum and, consequently, a displacement of the peaks to higher 2*θ* values, as observed in some studies reported in literature [15, 29, 34, 35].

The insertion of a metal in the other metal structure can form a substitutional or interstitial solid solution [36]. The face-centered cubic structure of Pt has two interstitial sites: one octahedral and the other tetrahedral. The relation between the radius of the octahedral (*r*_O_) and tetrahedral (*r*_T_) sites with atomic radius of the crystal lattice (*R*) is given by *r*_O_ = 0.41*R* and *r*_T_ = 0.22*R*, respectively. Considering the cubic structure of Pt and a metallic platinum radius of 139 pm, the radii of the octahedral and tetrahedral sites are 57 pm and 31 pm, which are smaller than metallic gallium radius (122 pm) [37]. Therefore, the formation of an interstitial solid solution does not occur. Although electroaffinity difference between theses metals (2.13 eV for Pt and 0.43 eV for Ga) and the difference between the structures (FCC for Pt and orthorhombic for Ga) can lead to formation of a new phase, the difference of 8% between the atomic radii (Pt, 139 pm, and Ga, 122 pm) and the same valence state of these metals does not rule out the formation of substitutional solid solution.

Average crystallite size ranging from 7.2 to 12.9 nm was observed for all the electrocatalysts, which matches the amounts presented for electrocatalysts synthesized from thermal decomposition of the polymeric precursor method [15, 19, 28, 29]. However, one observed that a few grew the crystallite size for the (111) diffraction plane, for all the electrocatalysts, except Pt_54_Ga_46_/C, which showed higher crystallite size value in the (222) diffraction plane. All electrocatalysts containing gallium showed smaller crystallite size than Pt_100_/C; therefore gallium addition causes crystallite size reduction and a substantially radial growth in relation to different planes. The electrooxidation of alcohols is influenced by the orientation of crystal plane of the platinum catalyst, since the reaction of platinum single crystals involves different steps of adsorption, resulting from interactions of adsorbed species with the metal surface direction. In a theoretical study of DFT, Wang and Liu [38] explained the location of the transition state for most surface reactions during the EOR on different surfaces of platinum planes, (111), (211), and (100). These results suggest that the Pt (100) is the best surface to completely oxidize ethanol in CO_2_ at low coverage; the other two planes are blocked from poisoning of intermediates which are difficult to decompose: acetate in the Pt (111) and CHCO in Pt (211). Acetaldehyde is the main product in the (111) formed in one concerted step of dehydrogenation of ethanol, while acetic acid is prevalent only in oxidative conditions such as presence of OH groups at high potential (>0.8 V). In this context, it is expected that acetaldehyde is the major product of ethanol oxidation in PtGa/C electrocatalysts, since they have slight increase toward the (111) plane, except for Pt_54_Ga_46_/C, which has radial growth. As the gallium content increases in the electrocatalyst, particle size in the (111) plane decreases. Therefore, the PtGa/C electrocatalyst may be more selective for the formation of more oxidized species such as acetic acid and CO_2_ compared with Pt_100_/C. According to Kumar et al. [26] the addition of gallium may modify the center of the d-band of the Pt metal. The d-band center shifts up to the Fermi level as compared to the parent metals via combination with a second metal over layering the Pt surface, resulting in an increase in the absorbate binding energy and thus promoting the performance of intermetallic for the CO oxidation.

**Figure 4:**
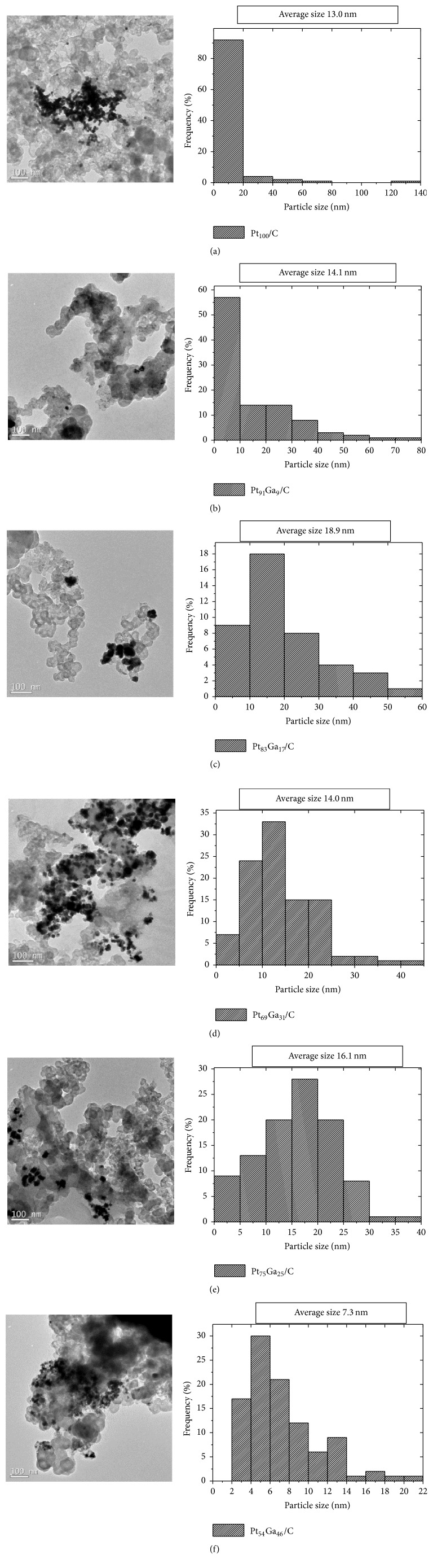
TEM images by side of particle size distribution of the PGa/C catalysts: (a) Pt_100_/C, (b) Pt_91_Ga_9_/C, (c) Pt_83_Ga_17_/C, (d) Pt_69_Ga_31_/C, (e) Pt_75_Ga_25_/C, and (f) Pt_54_Ga_46_/C.

The TEM images and the particle size distribution of PtGa/C electrocatalysts prepared by thermal decomposition of polymeric precursors supported on carbon Vulcan XC-72 are shown in Figure 4. All the electrocatalysts show heterogeneous dispersion of carbon support, a characteristic associated with the synthesis method [39]; an approximate circular shape and the particle size distribution show that as the quantity of gallium in the electrocatalyst increases there is less asymmetrical distribution of the population sizes, with average values between 7.3 and 18.9 nm. These values are higher than the values obtained from XRD (7.2 to 12.9 nm) and indicate the formation of small nanoparticle clusters.

### 3.2. Electrochemical Characterizations

**Figure 5:**
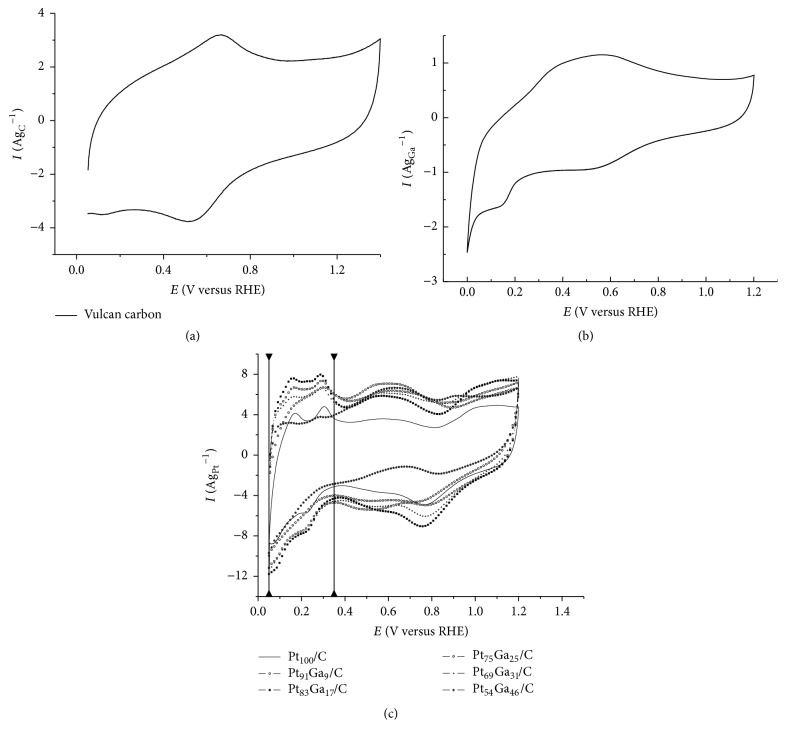
Cyclic voltammograms of the (a) Vulcan carbon XC-72; (b) Ga/C; and (c) PtGa/C electrocatalysts in H_2_SO_4_ 0.5 mol L^−1^ supporting electrolyte, from 0.05 to 1.2 V versus RHE and scan rate of 50 mV s^−1^.

Figure 5 shows the cyclic voltammograms (CV) obtained for Vulcan carbon/C (Figure 5(a)), Ga/C (Figure 5(b)), and different PtGa/C electrocatalysts in acid medium 0.5 mol L^−1^ H_2_SO_4_ (Figure 5(c)). As can be observed the Vulcan carbon/C electrode presents a behavior characteristic of carbon electrode. In the case of the Ga/C it is possible to observe a decrease in the value of the anodic and cathodic current in relation to the Vulcan carbon/C electrode; this suggests an inhibition of the electrode surface. On the other hand, a broadening of the anodic and cathodic peaks of the Ga/C electrode is visible, which may be related to redox transitions of gallium oxide. Further evidence is the emergence of a cathodic charge within potential region 0.0–0.2 V versus RHE, which also suggests a reduction of gallium oxide, perhaps from Ga^3+^ to Ga^0^.

Higher current densities for all compositions of PtGa/C electrocatalysts are observed compared to the pure platinum catalyst supported on carbon, except for the binary Pt_54_Ga_46_/C electrocatalyst, which has lower current density in hydrogen desorption region (0.05 to 0.35 V versus RHE). It is also visible that this region is distorted for all the electrocatalysts in comparison to pure platinum, which is related to the carbon support in the case of Pt_100_/C electrocatalyst and gallium and their oxides in the catalytic surface to the PtGa/C electrocatalyst. The electrocatalysts with highest experimental load of gallium, namely, the Pt_54_Ga_46_/C and the Pt_69_Ga_31_/C, showed the peak hydrogen desorption even less defined than the electrocatalysts with less gallium (e.g., Pt_75_Ga_25_/C, Pt_83_Ga_17_/C, and Pt_91_Ga_9_/C).

Due to the presence of gallium, oxides, and carbon on the catalyst surface, calculating the electrochemical active surface area (EASA) is a difficult task. However, it was estimated for each electrocatalyst by integrating the hydrogen desorption region (positive current) of the respective voltammograms in an acid medium in the range potential from 0.05 to 0.35 V versus RHE, obtaining the charge density into the process considering that 0.21 mC cm^−2^ represents the charge required to oxidize a monolayer of hydrogen on bright Pt [40] as presented in Table 3. The contribution of capacitive current due to the double layer capacitance does not have to be subtracted because it is not possible to make such subtraction without making a big mistake. The addition of up to 25% of gallium in the PtGa/C electrocatalyst increases the EASA from 11.4 to 33.4 m^2^ g_Pt_^−1^; however, for a higher gallium loading there is a continuous decrease of the EASA, 33.4 to 13.5 m^2^ g_Pt_^−1^, as observed for Pt_54_Ga_46_/C electrocatalyst. Therefore, the optimal composition for the PtGa/C electrocatalyst is placed between 9% and 31% of gallium loading.

**Figure 6:**
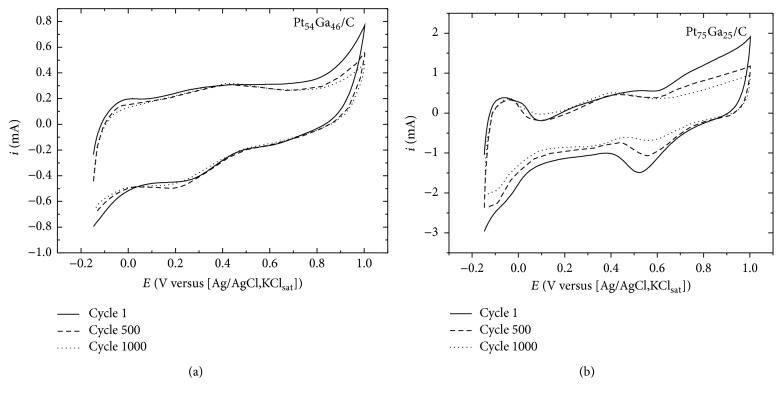
Representative CV of the PtGa/C electrocatalyst in H_2_SO_4_ 0.5 mol L^−1^ supporting electrolyte, from −0.15 to 1.0 V versus Ag/AgCl, KClsat, and scan rate of 20 mV s^−1^. (a) Pt_54_Ga_46_/C; (b) Pt_75_Ga_25_/C.

To investigate the electrocatalyst stability, test with 1000 cycles' voltammetry was conducted for all electrocatalysts; Figure 6 shows the representative behavior observed for the PtGa/C electrocatalysts. The results indicate that after 1000 cycles the electrocatalyst presents a slight decrease in both anodic and cathodic charge for all the electrocatalysts investigated. However, one made ICP-OES analysis of the supporting electrolyte after CV (1000 cycles), and the results showed few amount of metals (Pt and Ga) in the solution. This behavior indicates the higher electrocatalyst stability. Although there is dissolving, this is natural because it is catalytic material and degradation will occur as it is used, as is well known in the literature about the instability of Pt-based catalysts [15, 28]. The metal concentrations obtained in this investigation are presented in Table 3.

**Figure 7:**
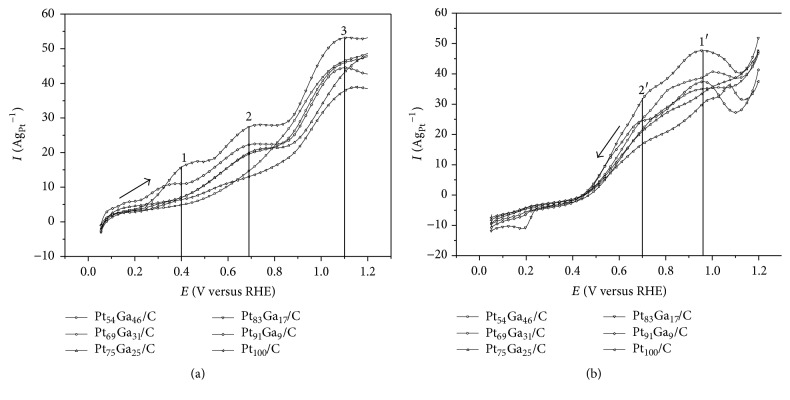
CV curves in the (a) positive-going scan and (b) negative-going scan for ethanol oxidation of the PtGa/C electrocatalysts in 1.0 mol L^−1^ ethanol and H_2_SO_4_ 0.5 mol L^−1^ supporting electrolyte, from 0.05 to 1.2 V versus RHE and scan rate of 50 mV s^−1^.

The CV curves of the PtGa/C electrocatalysts obtained in 1.0 mol L^−1^ of ethanol in supporting electrolyte (0.5 mol L^−1^ H_2_SO_4_) are shown in Figures 7(a) and 7(b). The current density values in the hydrogen desorption region are smaller compared to the CV curves of Figure 5, which could be associated with adsorption of ethanol molecules on platinum sites. The CV in ethanol is divided as follows: in the positive-going scan (Figure 7(a)), the Pt_91_Ga_9_/C and Pt_83_Ga_17_/C electrocatalysts show three peaks (1, 2, and 3), Pt_69_Ga_31_/C and Pt_75_Ga_25_/C have two peaks (2 and 3), Pt_54_Ga_46_/C and Pt_100_/C have only one peak (3). In the negative-going scan (Figure 7(b)), all electrocatalysts exhibit two peaks (1′ and 2′).

These peaks could be interpreted as the oxidation of different organic species adsorbed on the catalyst surface. According to studies of Wang and coworkers [41], the initial oxidation of ethanol to acetaldehyde at low potential (<0.4 V versus RHE) is often difficult due to blockage of the catalyst with CO and adsorbed carbonaceous compounds. In Figure 7(a) the Pt_91_Ga_9_/C and Pt_83_Ga_17_/C electrocatalysts are the ones to present well defined peaks in this region as well as earlier onset potential for EOR; other electrocatalysts show a linear current increase. In peaks 2 and 3, in Figure 7(a), current density increases considerably in the PtGa/C electrocatalysts compared to the pure Pt/C electrocatalyst, which may be related to the continued oxidation of ethanol to acetaldehyde and acetic acid formation at higher potentials, according to the following reaction:

(3) $$\begin{array}{c} {CH}_{3}{CH}_{2}OH+\frac{1}{2}H_{2}O⟶\\ \frac{1}{2}{CH}_{3}COOH+\frac{1}{2}{CH}_{3}CHO+3H^{+}+3e^{-} \end{array}$$

In the negative-going scan (Figure 7(b)) one can also observe that the PtGa/C electrocatalysts promote the oxidation of compounds adsorbed on the surface of the electrocatalyst and that there is presence of two resolved peaks (1′ and 2′). According to studies of Wang and coworkers [41], products as methane, ethane, acetic acid, ethyl acetate, or even acetaldehyde were identified with platinum electrocatalyst supported on carbon in similar reaction conditions through mass spectroscopy experiments.

**Figure 8:**
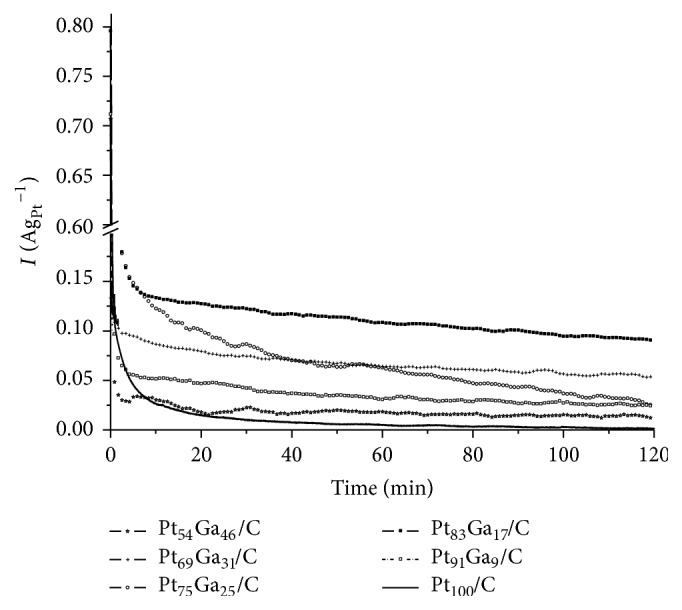
Current versus time plots for the electrooxidation of 1.0 mol L^−1^ ethanol in H_2_SO_4_ 0.5 mol L^−1^ at 0.4 V versus RHE on various PtGa/C electrocatalysts.

After the voltammetric analysis in the presence of alcohol, the PtGa/C electrocatalysts were submitted to experiments at fixed potential of 0.4 V versus RHE for two hours (chronoamperometry, CA). The results of chronoamperometry are shown in Figure 8. It is observed that the addition of any amount of gallium to the platinum electrocatalyst promotes an increase of the ethanol oxidation current. In the initial minutes of analysis, there is an abrupt current value decline due to the charging of the catalytic surface and a slight decrease of the current value is observed as a function of time, which is associated with inactivation of electrocatalytic sites due to irreversible desorption of intermediates (CO, CH_x_, and CH_3_CHO) [15, 19]; thus a good electrocatalyst will be the one with higher removal ability of these adsorbed species.

Pt_83_Ga_17_/C and Pt_75_Ga_25_/C have the best results for EOR because they have higher and more stable current over time, while Pt_91_Ga_9_/C and Pt_54_Ga_46_/C have intermediate values and Pt_69_Ga_31_/C suffered abrupt decline in current during the analysis, indicating instability. One proposed explanation for this observation is that no significant amount of inclusion of gallium in the platinum structure, in the case of Pt_91_Ga_9_/C, and the addition above 25% of gallium might increase the formation of gallium oxides that maybe block the platinum surface.

To investigate the electrocatalyst stability, one also made ICP-OES analysis of the supporting electrolyte after CA, and the results showed that the amount of metals (Pt and Ga) in the solution was below limit of detection (LOD) and/or limit of quantification (LOQ), that is, LD Ga = 4.54 *µ*g/L and Pt = 20.6 *µ*g/L; LOQ Ga = 45.4 *µ*g/L and Pt = 206.4 *µ*g/L, with exception of the Pt_54_Ga_46_/C electrocatalyst that showed Ga concentration equal to 84.8 *µ*g/L.

**Figure 9:**
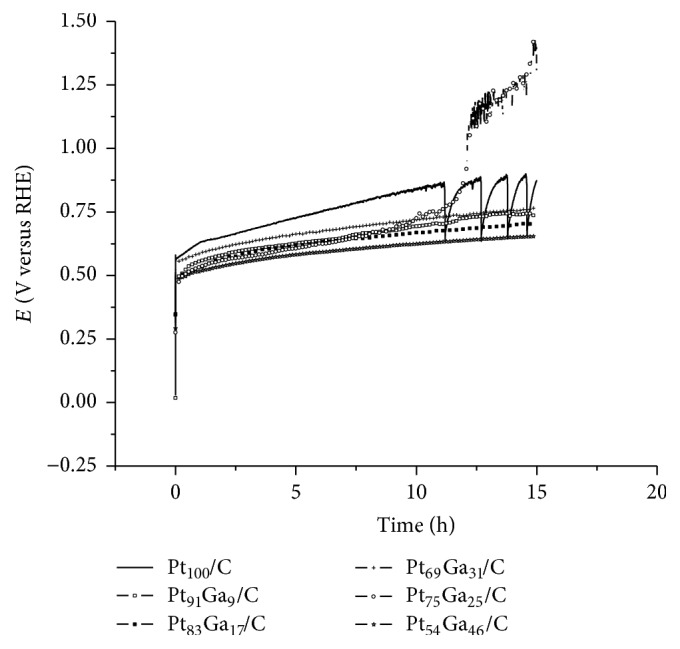
Chronopotentiometry of the PtGa/C electrocatalysts in 1.0 mol L^−1^ ethanol and H_2_SO_4_ 0.5 mol L^−1^ supporting electrolyte, with current density applied of 3.0 mA cm^−2^.

Chronopotentiometric curves (CP) are shown in Figure 9; the purpose of this experiment was to evaluate the stability of the electrocatalysts after the application of current density of 3.0 mA cm^−2^ for 15 hours. In the first hours of the experiment, the ethanol oxidation potential increases as the poisoning of catalytic sites occurs with CO and adsorbed intermediates, indicating that most stable catalysts have the lowest potential changes. The difference in values between initial and final potential is shown in Table 4. PtGa/C electrocatalysts have lowest potential changes during the CP experiment compared to the Pt_100_/C, indicating that the addition of gallium increases stability of the catalysts, except for Pt_75_Ga_25_/C which increased the potential and presented oscillatory behavior. The oscillatory behavior of the potential in CP on electrooxidation of small organic molecules such as methanol, ethanol, and formic acid on platinum catalysts is common and has already elucidated mechanism [42]. Platinum surface is poisoned by adsorbed CO in ethanol oxidation. In order to maintain the current, the potential increases to a point where the surface is completely renewed and the oscillatory behavior restarts. Higher oscillation amplitudes are related to low electrocatalytic activity for the electrooxidation of CO adsorbed. When catalysts have oscillatory behavior, the increase in potential can cause material degradation, metal dissolution, and carbon support corrosion [42]. Oscillation of potential of Pt_100_/C indicates an increase in CO concentration after 11 hours of experiment leading to poor electrocatalytic activity, while the Pt_75_Ga_25_/C showed a higher frequency of the oscillations after 12 hours and instability with abrupt increase in potential.

**Figure 10:**
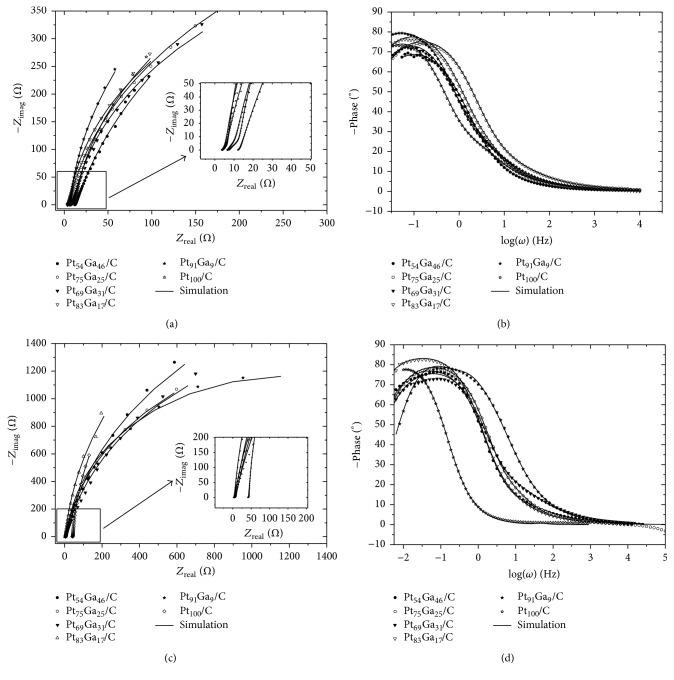
Nyquist ((a) and (c)) and Bode ((b) and (d)) plots of the PtGa/C electrocatalysts in ((a) and (b)) ethanol 1.0 mol L^−1^ in supporting electrolyte solution and ((c) and (d)) only supporting electrolyte.

The Nyquist plots and the Bode plots of EIS for PtGa/C electrocatalysts are depicted in Figure 10. The EIS data suggest that EOR shows different impedance behavior depending on the electrocatalyst composition. The size of the EIS arc and its slope yields information about the charge-transfer resistance of the reaction that takes place in the electrocatalyst. In Figure 10(a), the EIS at 0.4 V versus RHE exhibits large arcs for Pt_54_Ga_46_/C and Pt_69_Ga_31_/C electrocatalyst in comparison with the Pt_100_/C, Pt_91_Ga_9_/C, Pt_83_Ga_17_/C, and Pt_75_Ga_25_/C electrocatalysts, revealing a slower rate for ethanol oxidation reaction [43]. The highest ethanol charge-transfer resistance observed indicates that the electrocatalyst surface might be blocked by adsorbed ethanol species from C-C rupture and/or from ethanol dehydrogenation [44]. Regarding Pt_100_/C, Pt_91_Ga_9_/C, Pt_83_Ga_17_/C, and Pt_75_Ga_25_/C electrocatalysts, it can be noted that the Pt_83_Ga_17_/C and Pt_75_Ga_25_/C electrocatalysts exhibit larger arc than Pt_100_/C and Pt_91_Ga_9_/C electrocatalysts. However, the slope for Pt_91_Ga_9_/C and Pt_100_/C arcs is slightly greater than the slope for Pt_83_Ga_17_/C and Pt_75_Ga_25_/C electrocatalysts. This analysis suggests that Pt_91_Ga_9_/C and Pt_100_/C electrocatalysts show slower rate for EOR than Pt_83_Ga_17_/C and Pt_75_Ga_25_/C electrocatalysts. The equivalent circuit suggests a system that consists of electrode coated by two superimposed porous layers [45]. The EIS behavior of PtGa/C in presence of ethanol 1.0 mol L^−1^ is consistent with a carbon porous electrode already reported in the literature [46, 47].

[*R*1(*R*2[*Q*1*W*1])] was simulated to estimate the electrical properties of PtGa/C electrocatalysts, the *R*1 resistance is related to the solution resistance regarding the pore distribution, *R*2 is the charge-transfer resistance inside the porous electrode, and, finally, *Q*1 and *W*1 are a constant phase element, which is related to the pore size distribution, and a Warburg impedance due to the ethanol diffusion process, respectively. The fitted charge-transfer resistance (see Table 5) agrees with chronoamperometry data, which indicates that the Pt_83_Ga_17_/C (1101 Ω) and Pt_75_Ga_25_/C (1056 Ω) are better PtGa/C electrocatalysts for EOR.

## 4. Conclusion

The TGA analysis helped to set the calcination temperature of the electrocatalysts and the EDX data showed good relationship between the nominal and the experimental composition for all electrocatalysts except for the Pt_69_Ga_31_/C, which has lost gallium during calcination. TEM images and XRD data showed that the particle sizes of electrocatalysts were ranging from 7.2 to 12.9 nm. The XRD results showed that the Pt was obtained with face-centered cubic structure and possibly formation of solid solution between gallium and platinum. EDX data also evidenced the possible presence of Pt and/or Ga oxides due to the great oxygen amounts in the electrocatalysts. The onset potential for the ethanol oxidation was ~0.30 V versus RHE for the Pt_83_Ga_17_/C and Pt_91_Ga_9_/C electrocatalysts. The EOR current values observed for PtGa/C electrocatalysts indicate that activation takes place at the electrocatalysts surface, which can be associated with the added gallium. Furthermore, the development of PtGa/C electrocatalyst is promising because it has higher and more stable current response as compared to pure platinum electrocatalyst mainly when the addition of gallium is up to 31% gallium loading.

## Figures and Tables

**Table 1:** EDX results and compositions of PtGa/C electrocatalysts.

Nominal composition (% mol)	Experimental composition (% mol)	Nominal weight (mg)	Experimental weight (mg)	Weight loss Pt : Ga (%)	Elemental composition of catalyst (wt.%) Pt : Ga			
					Platinum	Gallium	Oxygen	Carbon
100 : 0	100 : 0	30	19.2	36	35.0	0.0	3.4	61.6
90 : 10	91 : 9	50	46.2	7.6	26.6	4.7	4.7	66.0
80 : 20	83 : 17	50	44.4	11.2	25.4	5.2	5.6	63.8
70 : 30	75 : 25	50	44.8	10.4	23.6	10.7	6.8	59.0
60 : 40	69 : 31	50	42.2	15.6	14.7	4.9	6.5	73.8
50 : 50	54 : 46	50	44.1	11.8	17.5	10.2	10.3	62.0

**Table 2:** Lattice parameter and volume and average crystallite size in the different diffraction planes of the PtGa/C electrocatalysts.

Experimental composition (% mol)	*a* (Å)	*V* (Å^3^)	*d* (nm)				
			111	200	220	311	222
Pt_100_	3.922	60.33	12.9	11.8	10.9	10.1	11.0
Pt_91_Ga_9_	3.922	60.32	11.5	10.6	9.7	9.1	10.3
Pt_83_Ga_17_	3.919	60.19	10.7	9.4	9.0	8.7	9.7
Pt_69_Ga_31_	3.906	59.58	10.4	9.2	8.9	8.3	9.6
Pt_83_Ga_17_	3.909	59.75	11.2	9.8	8.9	8.3	8.9
Pt_54_Ga_46_	3.907	59.64	8.5	8.0	7.6	7.2	8.7

**Table 3:** Hydrogen desorption/adsorption charge, EASA, and [Ga] of the PtGa/C electrocatalysts.

Electrocatalyst	Hydrogen desorption charge (Cg_Pt_^−1^)	Hydrogen adsorption charge (Cg_Pt_^−1^)	EASA^**∗**^ (m^2^g_Pt_^−1^)	[Ga] (mgL^−1^)
Pt_100_/C	24.0	39.8	11.4	0.00^*∗∗*^
Pt_91_Ga_9_/C	51.5	64.5	24.5	1.19
Pt_83_Ga_17_/C	56.6	67.2	27.0	0.87
Pt_75_Ga_25_/C	70.1	100.0	33.4	3.66
Pt_69_Ga_31_/C	41.5	55.3	19.8	2.03
Pt_54_Ga_46_/C	28.4	52.3	13.5	1.56

^*∗*^EASA = electrochemical active surface area; ^*∗∗*^[Pt] = 0.041 mg/L.

**Table 4:** Potential variation of PtGa/C electrocatalysts after 15 hours of electrolysis in ethanol 1.0 mol L^−1^ at 3.0 mA cm^−2^.

Electrocatalyst	*E*_I_ (V versus RHE)	*E*_F_ (V versus RHE)	*E*_F_–*E*_I_ (V versus RHE)
Pt_100_/C	0.56	0.87	0.31
Pt_91_Ga_9_/C	0.50	0.74	0.24
Pt_83_Ga_17_/C	0.50	0.70	0.20
Pt_69_Ga_31_/C	0.55	0.76	0.21
Pt_75_Ga_25_/C	0.50	1.28	0.78
Pt_54_Ga_46_/C	0.47	0.65	0.18

**Table 5:** Fitted circuit [*R*1(*R*2[*Q*1*W*1])] parameters of the PtGa/C electrocatalysts^*∗*^.

Circuit elements/unit	Pt_100_	Pt_91_Ga_9_/C	Pt_83_Ga_17_/C	Pt_75_Ga_25_/C	Pt_69_Ga_31_/C	Pt_54_Ga_46_/C

In presence of ethanol						
*R*1/Ω	7.18	3.83	3.64	6.44	3.92	11.95
*R*2/Ω	1194	1630	1025	1030	1212	1570
*Q*1/mF	12.99	17.07	13.1	8.42	4.64	1.94
*n *	0.95	0.96	0.95	0.94	0.92	0.88
*W*1/Ω	5.44	2.65	1.89	4.23	1.85	1.32

In absence of ethanol						
*R*1/Ω	41.46	2.68	2.9	3.9	3.9	5.9
*R*2/Ω	5641	2387	5343	3455	3106	4334
*Q*1/mF	16.4	8.8	18,1	3.8	6.30	4.32
*n*	0.97	0.99	0.96	0.90	0.92	0.909
*W*1/Ω	1.85	15.8	1.40	3.2	1.3	2.2

^*∗*^Chi-square observed for all simulation ranged from 10^−4^ to 10^−5^ and the error for the data values simulation was obtained between 0.08 and 15%. *Q*1 is constant phase element; *R*1 is ohmic resistance; *R*2 is charge transfer resistance; *W*1 is Warburg impedance.

## References

[1] Wang Y., Chen K. S., Mishler J., Cho S. C., Adroher X. C. (2011). A review of polymer electrolyte membrane fuel cells: technology, applications, and needs on fundamental research. *Applied Energy*.

[2] Brouzgou A., Song S. Q., Tsiakaras P. (2012). Low and non-platinum electrocatalysts for PEMFCs: current status, challenges and prospects. *Applied Catalysis B: Environmental*.

[3] Moreira J. R., Pacca S. A., Parente V. (2014). The future of oil and bioethanol in Brazil. *Energy Policy*.

[4] Ajanovic A., Haas R. (2014). On the future prospects and limits of biofuels in Brazil, the US and EU. *Applied Energy*.

[5] Badwal S. P. S., Giddey S., Kulkarni A., Goel J., Basu S. (2015). Direct ethanol fuel cells for transport and stationary applications—a comprehensive review. *Applied Energy*.

[6] Simões M., Baranton S., Coutanceau C. (2010). Electro-oxidation of glycerol at Pd based nano-catalysts for an application in alkaline fuel cells for chemicals and energy cogeneration. *Applied Catalysis B: Environmental*.

[7] Figueiredo M. C., Sorsa O., Doan N. (2015). Direct alcohol fuel cells: increasing platinum performance by modification with sp-group metals. *Journal of Power Sources*.

[8] Liu Z., Ma L., Zhang J., Goodwin J. G. (2013). Pt alloy electrocatalysts for proton exchange membrane fuel cells: a review. *Catalysis Reviews*.

[9] Zheng Q., Cheng X., Jao T.-C., Weng F.-B., Su A., Chiang Y.-C. (2012). Degradation analyses of Ru_85_Se_15_ catalyst layer in proton exchange membrane fuel cells. *Journal of Power Sources*.

[10] Almeida T. S., Kokoh K. B., de Andrade A. R. (2011). Effect of Ni on Pt/C and PtSn/C prepared by the Pechini method. *International Journal of Hydrogen Energy*.

[11] Oliveira Neto A., Dias R. R., Ribeiro V. A., Spinacé E. V., Linardi M. (2006). Eletro-oxidação de etanol sobre eletrocatalisadores PtRh/C, PtSn/C e PtSnRh/C preparados pelo método da redução por álcool. *Eclética Química*.

[12] Colmati F., Antolini E., Gonzalez E. R. (2006). Effect of temperature on the mechanism of ethanol oxidation on carbon supported Pt, PtRu and Pt_3_Sn electrocatalysts. *Journal of Power Sources*.

[13] Tayal J., Rawat B., Basu S. (2011). Bi-metallic and tri-metallic Pt-Sn/C, Pt-Ir/C, Pt-Ir-Sn/C catalysts for electro-oxidation of ethanol in direct ethanol fuel cell. *International Journal of Hydrogen Energy*.

[14] Lopes T., Antolini E., Colmati F., Gonzalez E. R. (2007). Carbon supported Pt–Co (3:1) alloy as improved cathode electrocatalyst for direct ethanol fuel cells. *Journal of Power Sources*.

[15] Ribeiro J., dos Anjos D. M., Kokoh K. B. (2007). Carbon-supported ternary PtSnIr catalysts for direct ethanol fuel cell. *Electrochimica Acta*.

[16] Teran F. E., Santos D. M., Ribeiro J., Kokoh K. B. (2012). Activity of PtSnRh/C nanoparticles for the electrooxidation of C1 and C2 alcohols. *Thin Solid Films*.

[17] Caliman C. C., Palma L. M., Ribeiro J. (2013). Evaluation of Ni and Ti addition in PtSn/C catalysts for ethanol and glycerol electrooxidation. *Journal of the Electrochemical Society*.

[18] Song S. Q., Zhou W. J., Zhou Z. H. (2005). Direct ethanol PEM fuel cells: the case of platinum based anodes. *International Journal of Hydrogen Energy*.

[19] Cunha E. M., Ribeiro J., Kokoh K. B., De Andrade A. R. (2011). Preparation, characterization and application of Pt-Ru-Sn/C trimetallic electrocatalysts for ethanol oxidation in direct fuel cell. *International Journal of Hydrogen Energy*.

[20] Vasconcellos M. L. (2014). A teoria de pearson para a disciplina de química orgânica: um exercício prático e teórico aplicado em sala de aula. *Química Nova*.

[21] Pandey B., Cox C. B., Thapa P. S., Ito T. (2014). Potentiometric response characteristics of oxide-coated gallium electrodes in aqueous solutions. *Electrochimica Acta*.

[22] Pandey B., Thapa P. S., Higgins D. A., Ito T. (2012). Formation of self-organized nanoporous anodic oxide from metallic gallium. *Langmuir*.

[23] Wei C., Bard A. J., Kapui I., Nagy G., Toth K. (1996). Scanning electrochemical microscopy. 32. Gallium ultramicroelectrodes and their application in ion-selective probes. *Analytical Chemistry*.

[24] Zhao Z., Yang Y., Xiao Y., Fan Y. (2012). Recovery of gallium from Bayer liquor: a review. *Hydrometallurgy*.

[25] Hogarth M. P., Ralph T. R. (2002). Catalysis for low temperature fuel cells part III: challenges for the direct methanol fuel cell. *Platinum Metals Review*.

[26] Kumar V. B., Sanetuntikul J., Ganesan P., Porat Z., Shanmugam S., Gedanken A. (2016). Sonochemical Formation of Ga-Pt Intermetallic Nanoparticles Embedded in Graphene and its Potential Use as an Electrocatalyst. *Electrochimica Acta*.

[27] Artem L. M., Santos D. M., De Andrade A. R., Kokoh K. B., Ribeiro J. (2012). Development of ternary and quaternary catalysts for the electrooxidation of glycerol. *The Scientific World Journal*.

[28] Evangelista T. C. S., Paganoto G. T., Guimarães M. C. C., Ribeiro J. (2015). Raman spectroscopy and electrochemical investigations of pt electrocatalyst supported on carbon prepared through plasma pyrolysis of natural gas. *Journal of Spectroscopy*.

[29] Purgato F. L. S., Olivi P., Léger J.-M. (2009). Activity of platinum-tin catalysts prepared by the Pechini-Adams method for the electrooxidation of ethanol. *Journal of Electroanalytical Chemistry*.

[30] Cullity B. D. (1956). *Elements of X Ray Diffraction*.

[31] Powder Diffraction File: 01-087-0646, Joint Committee on Powder Diffraction Standards Inter. Center for Diffraction Data, V. PDF-2, 2011

[32] Ristić M., Popović S., Musić S. (2005). Application of sol-gel method in the synthesis of gallium(III)-oxide. *Materials Letters*.

[33] Shirako Y., Wang X., Tsujimoto Y. (2014). Synthesis, crystal structure, and electronic properties of high-pressure PdF_2_-Type Oxides MO_2_ (M = Ru, Rh, Os, Ir, Pt). *Inorganic Chemistry*.

[34] Almeida T. S., Palma L. M., Leonello P. H., Morais C., Kokoh K. B., de Andrade A. R. (2012). An optimization study of PtSn/C catalysts applied to direct ethanol fuel cell: effect of the preparation method on the electrocatalytic activity of the catalysts. *Journal of Power Sources*.

[35] Bonesi A. R., Triaca W. E., Castro L. A. M. (2009). Nanocatalysts for ethanol oxidation. Synthesis and characterisation. *Portugaliae Electrochimica Acta*.

[36] Callister W. D. (2007). *Materials Science and Engineering: An Introduction*.

[37] Haynes W. M. (2009). *CRC Handbook of Chemistry and Physics*.

[38] Wang H. F., Liu Z. P. (2008). Comprehensive mechanism and structure-sensitivity of ethanol oxidation on platinum: new transition-state searching method for resolving the complex reaction network. *Journal of the American Chemical Society*.

[39] Spinacé E. V., Neto A. O., Franco E. G., Linardi M., Gonzalez E. R. (2004). Methods of preparation of metal nanoparticles supported on high surface area carbon as electrocatalysts in proton exchange membrane fuel cells. *Quimica Nova*.

[40] Zhang M., Yan Z., Li Y., Jing J., Xie J. (2015). Preparation of cobalt silicide on graphene as Pt electrocatalyst supports for highly efficient and stable methanol oxidation in acidic media. *Electrochimica Acta*.

[41] Wang H., Jusys Z., Behm R. J. (2004). Ethanol electrooxidation on a carbon-supported Pt catalyst: reaction kinetics and product yields. *Journal of Physical Chemistry B*.

[42] Nagao R., Cantane D. A., Lima F. H. B., Varela H. (2013). Influence of anion adsorption on the parallel reaction pathways in the oscillatory electro-oxidation of methanol. *Journal of Physical Chemistry C*.

[43] Bonesi A. R., Moreno M. S., Triaca W. E., Castro Luna A. M. (2010). Modified catalytic materials for ethanol oxidation. *International Journal of Hydrogen Energy*.

[44] Camara G. A., Iwasita T. (2005). Parallel pathways of ethanol oxidation: the effect of ethanol concentration. *Journal of Electroanalytical Chemistry*.

[45] Orazem M. E., Tribollet B. (2008). *Electrochemical Impedance Spectroscopy*.

[46] Song H., Jung Y., Lee K., Dao L. H. (1999). Electrochemical impedance spectroscopy of porous electrodes: the effect of pore size distribution. *Electrochimica Acta*.

[47] Yoo H. D., Jang J. H., Ryu J. H., Park Y., Oh S. M. (2014). Impedance analysis of porous carbon electrodes to predict rate capability of electric double-layer capacitors. *Journal of Power Sources*.

